# Sequential interim analyses of survival data in DNA microarray experiments

**DOI:** 10.1186/1471-2105-12-127

**Published:** 2011-04-29

**Authors:** Andreas Leha, Tim Beißbarth, Klaus Jung

**Affiliations:** 1 Department of Medical Statistics, University Medical Center Göttingen, D-37099 Göttingen, Germany

## Abstract

**Background:**

Discovery of biomarkers that are correlated with therapy response and thus with survival is an important goal of medical research on severe diseases, e.g. cancer. Frequently, microarray studies are performed to identify genes of which the expression levels in pretherapeutic tissue samples are correlated to survival times of patients. Typically, such a study can take several years until the full planned sample size is available.

Therefore, interim analyses are desirable, offering the possibility of stopping the study earlier, or of performing additional laboratory experiments to validate the role of the detected genes. While many methods correcting the multiple testing bias introduced by interim analyses have been proposed for studies of one single feature, there are still open questions about interim analyses of multiple features, particularly of high-dimensional microarray data, where the number of features clearly exceeds the number of samples. Therefore, we examine false discovery rates and power rates in microarray experiments performed during interim analyses of survival studies. In addition, the early stopping based on interim results of such studies is evaluated. As stop criterion we employ the achieved average power rate, i.e. the proportion of detected true positives, for which a new estimator is derived and compared to existing estimators.

**Results:**

In a simulation study, pre-specified levels of the false discovery rate are maintained in each interim analysis, where reduced levels as used in classical group sequential designs of one single feature are not necessary. Average power rates increase with each interim analysis, and many studies can be stopped prior to their planned end when a certain pre-specified power rate is achieved. The new estimator for the power rate slightly deviates from the true power rate but is comparable to other estimators.

**Conclusions:**

Interim analyses of microarray experiments can provide evidence for early stopping of long-term survival studies. The developed simulation framework, which we also offer as a new R package 'SurvGenesInterim' available at http://survgenesinter.R-Forge.R-Project.org, can be used for sample size planning of the evaluated study design.

## Background

A frequent objective of cancer related studies is to detect genes or biomarkers that can predict the outcome of therapy. The hardest criterion of success for therapies is the survival of patients. To identify predictive genes, the expression levels in samples of tumor or normal tissue are measured by DNA microarrays before the therapy is applied. Then, expression levels are compared to survival data of the patients. Usually, tissue samples are only available at distinguished points in time and it can take several years until the full planned sample size is available and follow-up is complete. In such long-lasting studies it would be beneficial to obtain interim results already before their planned end. An early detection of survival related genes within an interim analysis would for example allow their further laboratory validation before the end of the study. In addition, if an interim analysis provides evidence for the early stopping of the study it would save time and costs and would spare further patients to be involved or allow better treatment. Classically, interim analyses are performed in studies of group sequential designs. In such designs, interim analyses are performed when a certain fraction of the full planned sample size *N *has been reached, for example when 1/3 · *N *and 2/3 · *N *samples are available. There are numerous articles that deal with group sequential designs in the case of one single feature, e.g. [1,2]. As the repeated testing of the same hypothesis by interim analyses is one form of multiple testing and thus inflates the overall type I error [3], both articles propose reduced significance levels to solve this problem. DNA microarray data, however, comprise expression levels for thousands of genes, meaning that there are more features than available samples. For this case of high-dimensional data there have been very few approaches published to date. Marot and Mayer [4], for example, propose a method for combining *p*-values from several independent microarray analyses and show that the overall false discovery rate is not inflated when testing repeatedly a large number of hypothesis. A similar result was obtained by Posch et al. [5]. We make use of these results and apply them for studies in which survival data is correlated with gene expression data in interim analyses. In order to detect the survival related genes, we use gene-wise Cox-regression analyses [6].

An important issue in interim analyses of multiple features is the choice of a stopping criterion. In the case of testing one single feature, the study is simply stopped when a significant result is observed. Similarly, Victor and Hommel [7] use gene-wise stopping rules in the case of high-dimensional microarray data. Marot and Mayer [4] and Posch et al. [5] propose to stop the study when a certain proportion of true positive genes has been detected. We pick up the latter idea and derive an estimator for the proportion of true positive findings This new estimator is compared to a variant based on [8] and to an estimator proposed in [4].

The article is structured as follows. The study design, the detection of survival-related genes, the problem of performing interim analyses in the case of multiple hypothesis testing, and the stop criterion are detailed in the Methods section. Subsequently, a simulation study evaluates the behavior of the false discovery rate and of the estimator for the proportion of true positive detections. The simulation study covers several settings of survival-focused microarray experiments with interim analyses. After presenting the results from these simulations, we apply our methods to gene expression data from a breast cancer study van de Vijver et al. [9]. Parameters from this breast cancer study are used one more simulation presented subsequently. Finally, a discussion on the results follows, and further ideas are given.

## Methods

This section starts with an illustration of the particular study design we are considering in this article. As survival is a special focus of this work, we detail afterwards the methods for the detection of survival-related genes. Next, an overview of common methods for multiple hypothesis testing and their application in group sequential interim analyses is given. In this context, we also describe rules for the early stopping of such studies.

First, let us introduce the basic notation. Let *N *be the total number of subjects that would be involved if the study was not stopped after any interim analysis. For each of the relating tissue samples the expression levels of *d *genes are measured by means of DNA microarrays. We denote the complete (*d *× *N*) data matrix with all genes and all samples by **X**. As typical for microarray data, each row represents one gene and each column represents one sample. Thus, *x_ij _*denotes the expression level of gene *i *in the tissue sample of subject *j *(*i *= 1, ..., *d*; *j *= 1, ..., *N*). An overview of all notations is given in Table 6.

### Study Design

Assume, the whole study is not stopped after any interim analysis. Then, the *N *patients will have individual arrival times **a **= (*a*_1_, ..., *a_N _*)', e.g. months after the begin of the study. We denote the arrival time of the last patient to enter the study by *l*_1 _= *max*(**a**). Thus, the first part of the study, during which patients are recruited, will take place in the time frame [0, *l*_1_].

**Figure 1:**
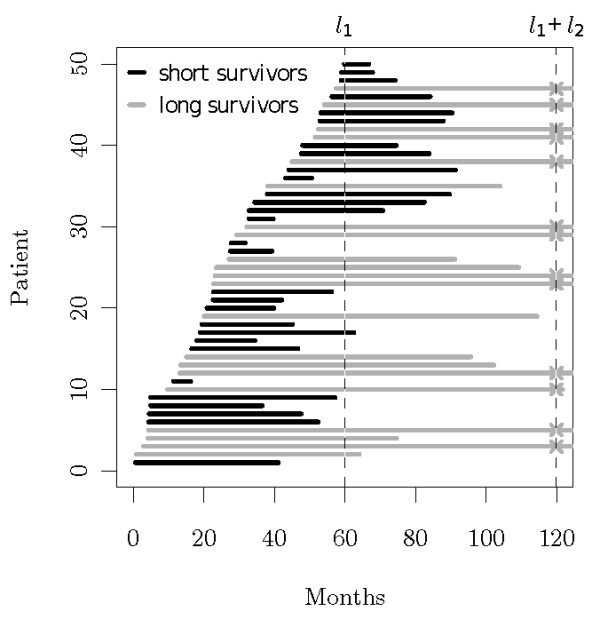
**Study Design**. The arrival time of each patient is marked by the left end of each horizontal bar and his death by the right end. The dashed lines represent the end of the recruitment part of the study and the end of the follow-up part, respectively. Patients marked with an 'x' have censored survival times at the final analysis.

Let us consider a second study episode which serves as a follow-up time of length *l*_2_, where no new patients enter the study but the patients survival data is still observed and up-dated. Thus, the length of the full study without any early stopping would be *L *= *l*_1 _+ *l*_2_, and the time frame of the second study part would be [*l*_1_, *L*]. The study design is visualized in Figure 1.

Assume further, that *M*_1 _interim analyses are planned to take place during the first study part. An interim analysis is always performed when (1/*M*_1_) · *N *new samples are available. This makes sure, that the sample size in each analysis is equal. In addition, *M*_2 _interim analyses are planned for the second study part, where an interim analysis is always performed when a time proportion of (1/*M*_2_) · *l*_2 _of the planned follow-up time has passed. Thus, we chose to perform analyses at equally spaced time-steps during this second part of the study. We denote the times at which interim analyses are performed by *t_m _*(*m *= 1, ..., *M*, where *M *= *M*_1 _+ *M*_2_).

### Detecting Survival-related Genes

Disease specific survival is the hardest control for measuring the success of cancer therapies. Therefore, we modelled the survival information in dependence on the gene expression data using Cox proportional hazard regression as was proposed for example by Simon et al. [10]. Survival information consists for each patient *j *of a pair (*s_j_*, *z_j_*), where *s_j _*is the observed survival time of the patient, and *z_j _*∈ {0, 1} specifies whether the patient has already died or not at the time the analysis takes place. According to the Cox-regression model, the survival information is modelled in terms of the hazard function at time *t *in dependence on gene *i *by(1)

where *h*_0 _is an unspecified function of *t*, called the baseline hazard. The hazard function *h*(*t*) can be interpreted as the patients risk of dying in a short time frame, [*t*, *t *+ *ε*), assuming the patient has survived thus far [11]. More precisely, the hazard function is defined as(2)

where t* is the patients observed survival time [12]. The influence of gene *i *on survival can be determined by testing the hypothesis *H*_0*i*_: *β_i _*= 0 in the related Cox model. The *d *resulting *p*-values from the gene-wise survival analyses can than be adjusted for multiple testing as described below.

In general, other models than the Cox model can be considered to detect survival-related genes. Park et al. [13] for example propose to use partial least squares regression to account for the presence of covariates.

### Interim Analyses of Multiple Endpoints

In each interim analysis one statistical test is performed per gene in order to detect those genes which correspond to the studied response variable (e.g. overall survival).

If the *d *hypotheses were all true and independent, testing each of them at the same level *α*, the expected number of false positive detections would be given by *α *· *d*. In whole genome microarray experiments, where *d *is typically about 40.000, the expected number of false positive detections would be too large to be tolerable. In multiple testing situations, it is therefore common to reduce the number of false positive decisions by controlling a pre-specified type I error rate. Note that the notion of *type I error rate *is not used consistently in the literature. Following Dudoit et al. [14] we will use the term *type I error rate *to name the superordinate concept of different types of such error rates, among which there are the *family-wise error rate (FWER) *and the *false discovery rate (FDR)*.

In microarray experiments the *FDR*, introduced by Benjamini and Hochberg [15], is the most commonly considered type I error rate. The *FDR *is defined as the expected proportion of false positives among all positive test decisions, i.e. *FDR *= *E*(*FP/R*), where *R *> 0 denotes the total number of rejected null-hypotheses. The proportion of false positives (FP) among all positives itself is also known as *false discovery proportion *(*FDP *= *FP*/*R*). In the special case that *R *= 0, i.e. no positive test decisions were found, the *FDP *as well as the *FDR *are defined to be zero.

The *FDR *can be controlled by adjusting the raw *p*-values resulting from the gene-wise tests. The adjusted *p*-values are then compared with a pre-specified level *α *of the *FDR *that is desired to be controlled. We denote the unadjusted *p*-value for gene *i *by *p_i _*and the respective adjusted *p*-value by . In our simulation study, we consider the adjusting procedure proposed by Benjamini and Hochberg [15]. Other adjusting procedures are detailed in [14].

Alternatively to comparing the adjusted *p*-values with the pre-specified *FDR*-level *α*, genes can be selected by comparing the raw *p*-values with adjusted *α*-levels. According to the procedure in [15], the raw *p*-values are ordered by increasing size, i.e. *p*_(1) _≤ *p*_(2) _≤ ... ≤ *p*_(*d*)_, and the largest *k *(*k *= 1, ..., *d*) for which *p*_(*k*) _≤ (*k*/*d*) *α *has to be determined. All hypothesis associated with *p*_(1) _to *p*_(*k*) _are then rejected. We denote the adjusted *α*-level that corresponds to this largest value of *k *by *α^BH^*.

Similar as in multiple hypothesis testing, the control of type I error rates is an important issue in group sequential interim analyses. In clinical trials on one single feature (for example when only one gene would be tested), interim analyses have been studied in-depth. As was shown by Armitage et al. [3], performing interim analyses increases the probability for making a type I error. In order to avoid such an increase and to maintain a pre-specified type I error, the tests at each interim analysis are performed at lower nominal levels  (*m *= 1, ..., *M*). The first authors who proposed *α*-level adjustments for group sequential interim analyses were Pocock [1] and O'Brien and Fleming [2].

At first glance, performing interim analyses in microarray experiments seems to require two adjustments. One to account for the interim analyses and one to account for multiple testing. However, the two recently published articles by Posch et al. [5] and by Marot and Mayer [4], show that the adjustment for interim analyses can be omitted, when *d *is large, while the *FDR *remains controlled. The result in [4] is based on the observation that the correlation between a single *p*-value and the empirical distribution of all *p*-values approaches zero when *d *gets large. The results in [5] are based on the findings of Storey et al. [16] who proved that, under certain assumptions,(3)

This holds for each interim analysis independently. Let  denote the random interim analysis where the study is stopped. Posch et al. [5] proved that equation (3) holds also at this interim analysis , since(4)

With equations (3) and (4) and the Lemma of Fatou it follows that the FDR is controlled asymptotically when *d *gets large.

This argumentation is not valid under the global null hypothesis (no gene significant), but it is also possible to extend the argumentation to the case of the global null hypothesis [5].

When performing interim analyses in experiments with multiple endpoints, one has to decide which data to base the interim analysis on. We decided to use all available accumulated data in each interim analysis.

This approach makes sure, that every analysis uses the maximal available data. However, one has to be aware of its drawback: it requires renormalization of the data in each analysis which leads to inconsistencies across the interim analyses.

### Stopping Rules

One important point in studies with planned interim analyses is the stop criterion. Each interim analysis provides the possibility to stop the study prior to its planned end, entailing the mentioned ethical and financial benefits. In studies on one single feature the study is usually stopped if that feature is found to be significant. In studies on a large number of features one could stop the study as soon as a pre-specified fixed number of features has been found to be significant. In the case of microarray analyses, this criterion might be useful when the number of genes that can be further validated by laboratory experiments is limited by costs or time.

Here, we follow the approach of Marot and Mayer [4] who consider as stop criterion the achieved proportion of detected true positives, the so called *average power rate (APR)*(5)

where *d*_1 _is the number of non-true null hypothesis. At each interim analysis the achieved *APR *is estimated and the study is stopped if this estimate exceeds a predefined level, e.g. 80%. In the case of *d*_1 _= 0 the *APR *is defined to be zero.

We employ a new estimator of the *APR*, similar to the *FDR*-estimator of Storey and Tibshirani [8] which is based on the following relations:(6)

The three components *E*[*R*], *E*[*FP*] and *E*[*d*_1_] can be estimated as in [8] which is shown in the following. The expectation of *R *can simply be estimated by the observed number of rejected hypothesis, i.e.(7)

The estimation of *E*[*FP*] is based on the fact, that *p*-values belonging to true null-hypotheses are uniformly distributed within [0,1]. Thus, the probability that a *p*-value which belongs to a true null-hypothesis is smaller than a threshold *t *(*t *∈ [0,1]) is exactly *t*. Therefore, if the significance level is chosen to be *α*' = *t *and *d*_0 _null hypotheses are true, *E*[*FP*] can be estimated by(8)

where *π*_0 _is the fraction of true null hypothesis. For *α' *one can for example choose *α^BH ^*as defined in the previous subsection. The unknown fraction *π*_0 _of true null hypotheses can be estimated by(9)

**Figure 2:**
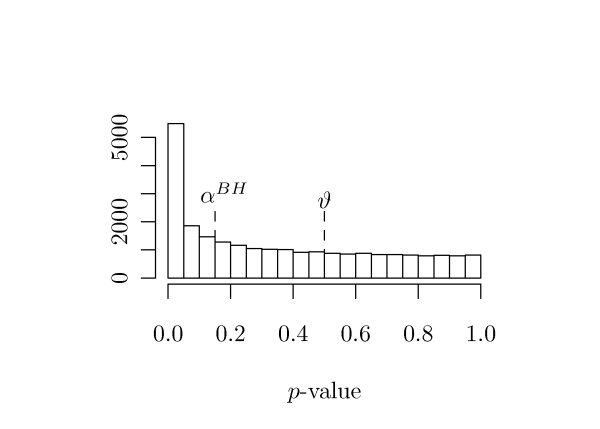
**Empirical *p*-Value Distribution from the Breast Cancer Data**. The tuning parameter *ϑ *is used within the estimation of the fraction *π*_0 _of true null hypothesis. The multiple testing adjusted *α*-level is marked with *α^BH^*.

Here, *ϑ *serves as a tuning parameter that balances bias versus variance. For a well chosen *ϑ*, the *p*-values in [*ϑ*, 1] will belong 'mostly' to true null-hypotheses, and therefore equation (9) estimates the fraction of true null-hypotheses. Again, the argument is the uniform distribution of *p*-values belonging to true null-hypotheses. See Figure 2 for graphical illustration.

According to our simulations (see below) setting *ϑ *= 0.5 results in a good estimate . Other automated ways to choose *ϑ *have been proposed in [17] and [8]. In both cases the estimate of *π*_0 _is used to estimate not the *APR *but the *FDR*. Storey [17] calculates the mean squared error of the *FDR *estimator for a range of values of *ϑ *and takes the one minimizing this MSE. The calculation of this MSE thereby is in turn based on a plug-in estimate of the *FDR*. The method presented in [8] does not need the *FDR *but is based on  only. The underlying observation is, that the bias in the estimator of *π*_0 _vanishes in the extreme choice *ϑ *= 1. Thus, the approach there is, to set .

A non-parametric estimator of *π*_0 _is given by Langaas et al. [18]. Based on this estimator and on the empirical distribution of the *p*-values Marot and Mayer [4] construct an *APR *estimator analog to the beta uniform model presented by [19].

In any case, the expectation of *d*_1 _can be estimated by(10)

such that the final estimator for the *APR *is given by(11)

We will use  to denote the estimator that results from setting *ϑ *= 0.5 and  (*S*) to denote the estimator which is based on the procedure for estimating *π*_0 _presented in [8]. The estimator by Marot and Mayer [4] will be denoted by  (*L*).

## Results

### Simulation Study

#### Data Generation and Settings

In order to simulate a study of the design proposed in subsection 'Study Design' we set the following parameters. At first, we chose the total number of samples to be *N *= 50. Furthermore, we set the intended length of the recruitment part of the study  and the length of the follow-up part to be 60 months, each, i.e. . We assume that the arrival times **a **are distributed uniformly during this first part. Thus, they were drawn from . The patients' survival times were assumed to follow an exponential distribution *Exp*(1/*λ*), where *λ *is the mean survival time. Here, we set *λ *= 60 months.

As we wanted to generate a set of genes which correspond to the simulated survival times, we split the individuals into two groups. The one group comprises the subjects with survival smaller than the specified *λ *the other group the subjects with equal or longer survival times than *λ*. The gene expression data for the two groups were then drawn from multivariate normal distributions *N_d_*(*μ_k_*, Σ), *k *= 1, 2. Expression levels were simulated for *d *= 10000 genes. The different mean vectors of the two groups represent the differentially expressed genes between subjects with short or long survival. For both groups, the same covariance matrix Σ with an autoregressive structure was generated. This structure images the fact that some genes are highly correlated among each other while others behave rather independent. In detail, we set(12)

**Figure 3:**
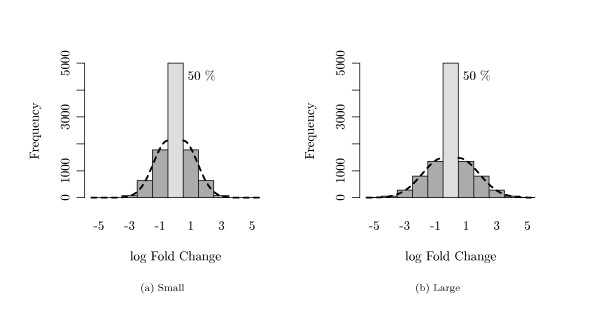
**Simulated Log Fold Changes**. Both plots display a distribution of log fold changes of the genes between short and long time survivors in the setting where 50% of all genes were not altered between both groups. Fold changes were drawn from a 'discretized' normal distribution (dashed line). (a) small fold changes, (b) large fold changes.

The expectation vector *μ*_1 _of the one group was set to be the null-vector, while a fraction of *τ *= 50% randomly chosen genes was altered in the other group. The alterations in *μ*_2 _were drawn from 'discretized' normal distributions. Larger fold changes were simulated via a higher standard deviation of this normal distribution. The structure of *μ*_2 _is illustrated in Figure 3.

This way we simulate an effect of a fixed size in the gene expression depending on whether the patient belongs to the group of long-term survivors or not. Of course in biology the inverse direction is true, i.e. survival is regulated by gene expression. However, we think that for our purpose it does not matter in which order the survival and expression data are generated. In addition, it is typical in biology that a gene is either up- or down-regulated. Hence, only the direction of regulation but not the strength of regulation influences the outcome. Therefore we chose to model the relationship between single genes and survival by a discrete function and not by some continuous one.

At each interim analysis, a gene-wise Cox regression was performed to detect the survival correlated genes, and resulting *p*-values were adjusted to control the *FDR *at a level of 5%. Following the results of Marot and Mayer [4] and of Posch et al. [5], no additional adjustment for interim analyses was performed. The number of simulation runs was set to 1000 for each setting. All simulations were performed with the free software R in version 2.10 [20].

We simulated two different setups. One, where 2 interim analyses are scheduled for both study parts, i.e. *M*_1 _= *M*_2 _= 2, and one where 5 analyses are planned to take place per part, i.e. *M*_1 _= *M*_2 _= 5. In each simulation run, our power estimator described in Section 'Stopping Rules' was applied and the study was stopped when an estimated *APR *of 80% was achieved.

As a more extreme setting we additionally simulated in the second setup (*M*_1 _= *M*_2 _= 5) the situation of a smaller fraction *τ *= 5% of altered genes.

#### Adherence to False Discovery Rate

**Figure 4:**
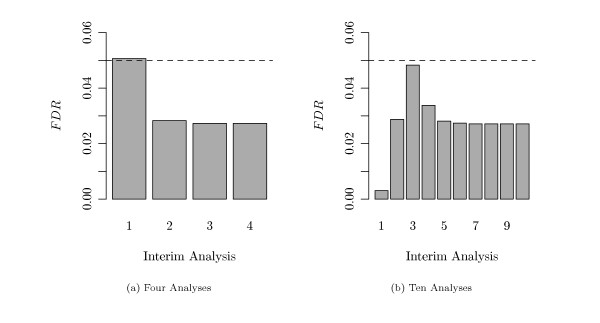
**Simulated *FDR *(*τ *= 50%)**. The simulated *FDR *is plotted at each interim analysis in the setting where 50% of all genes were altered between both groups. (a) *M *= 4 analyses (3 interim and 1 final), (b) *M *= 10 analyses. The dashed line marks the pre-specified *FDR*-level of 5%.

As no interim-specific adjustments were applied, the question arises, whether the pre-specified *FDR*-level of 5% was maintained at each analysis. Figure 4 displays the mean simulated *FDR *at each interim analysis in two different settings. Figure 4(a) represents the case of *M *= 4 analyses (i.e., 3 interim and one final), while Figure 4(b) represents the case of *M *= 10 analyses. Table 1 and Table 2 contain the mean and standard deviation of the *FDR *for these cases. Both results were obtained with the fold changes for the genes between long and short time survivors being generated as shown in Figure 3(b). The pre-specified *FDR*-level of 5%, indicated by the dashed line, is maintained at nearly each analysis.

**Figure 5:**
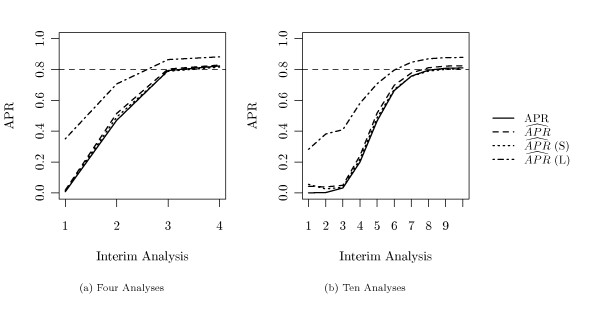
**Simulated Power Rate (*τ *= 50%)**. True (solid line) and estimated (broken lines) average power rates (*APR*) at each interim analysis in the setting where 50% of all genes were altered between both groups. (a) *M *= 4 analyses, (b) *M *= 10 analyses. The dashed line marks the pre-specified stopping criterion, i.e. an estimated *APR *of 80%.

**Table 1:** Simulated *FDR *and Power Rate (4 Analyses)

	**Analysis**							
	
	**1**		**2**		**3**		**4**	
				
	**mean **	**sd **	**mean **	**sd **	**mean **	**sd **	**mean **	**sd **
#found genes	38	180	2422	896	4079	379	4232	290
FDR	0.051	0.158	0.028	0.006	0.027	0.003	0.027	0.003
APR	0.007	0.035	0.471	0.174	0.793	0.074	0.823	0.056
	0.017	0.071	0.515	0.170	0.803	0.054	0.829	0.033
(S)	0.014	0.053	0.491	0.168	0.788	0.073	0.816	0.057
(L)	0.351	0.133	0.707	0.077	0.865	0.038	0.882	0.025

The descriptive values for each interim analysis including the number detected genes, the simulated *FDR*, the real *APR*, and the *APR *estimates, all in the setting of *τ *= 50% altered genes and four interim analyses. These values correspond to Figure 4 (a) and Figure 5 (a).

**Table 2:** Simulated *FDR *and Power Rate (10 Analyses)

	Analysis									
	
	1		2		3		4		5	
					
	mean	sd	mean	sd	mean	sd	mean	sd	mean	sd
#found genes	0	0	10	85	164	437	1033	928	2404	915
FDR	0.003	0.055	0.029	0.119	0.048	0.145	0.034	0.061	0.028	0.006
APR	0.000	0.000	0.002	0.016	0.032	0.085	0.200	0.180	0.467	0.178
	0.043	0.060	0.038	0.120	0.049	0.134	0.241	0.208	0.518	0.174
(S)	0.055	0.122	0.025	0.085	0.037	0.092	0.220	0.188	0.488	0.172
(L)	0.283	0.401	0.382	0.228	0.410	0.132	0.581	0.113	0.708	0.079

	**6**		**7**		**8**		**9**		**10**	
					
	**mean**	**sd**	**mean**	**sd**	**mean**	**sd**	**mean**	**sd**	**mean**	**sd**

#found genes	3414	637	3894	520	4092	471	4155	451	4167	446
FDR	0.027	0.004	0.027	0.004	0.027	0.004	0.027	0.004	0.027	0.004
APR	0.664	0.124	0.757	0.101	0.796	0.092	0.808	0.088	0.811	0.087
	0.698	0.096	0.778	0.062	0.812	0.043	0.823	0.034	0.825	0.031
(S)	0.671	0.117	0.756	0.098	0.790	0.091	0.802	0.087	0.804	0.086
(L)	0.797	0.056	0.847	0.042	0.870	0.031	0.877	0.025	0.878	0.023

The descriptive values for each interim analysis including the number detected genes, the simulated *FDR*, the real *APR*, and the *APR *estimates, all in the setting of *τ *= 50% altered genes with large fold changes and ten interim analyses. These values correspond to Figure 4 (b) and Figure 5 (b).

One can observe, that with only a small number of patients available the problem is harder, such that only a small number of genes is detected. In such cases each false positive gets more weight in the calculation of the *FDR *and one has to expect higher *FDR *levels. In later interim analyses, the simulated *FDR *stabilizes at a more conservative level. In the setting of *M *= 10 interim analyses, the *FDR *is considerably small in the very first interim analysis. This observation can be explained by the fact that the *FDR *is defined to be zero when no genes are found at all.

**Figure 6:**
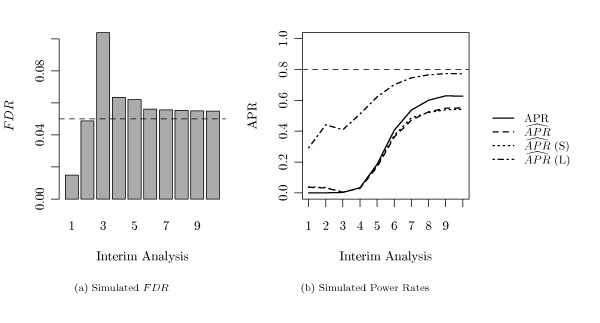
**Simulated *FDR *and Power Rate (*τ *= 5%)**. The simulated *FDR *(a) and the real *APR *(solid line) with the *APR *estimates (broken lines) (b) at each interim analysis in the setting where 5% of all genes were altered between both groups.

In the more extreme setting with only *τ *= 5% survival related genes, the overall course of the *FDR *over the interim analyses - as shown in Figure 6(a) and Table 3 - stays the same, but the peak in the early analyses becomes more prominent, and the specified *FDR*-level is not strictly maintained also during the later interim analyses.

**Table 3:** Simulated *FDR *and Power Rate (10 Analyses *τ *= 5%)

	Analysis									
	**1**		**2**		**3**		**4**		**5**	
					
	**mean**	**sd**	**mean**	**sd**	**mean**	**sd**	**mean**	**sd**	**mean**	**sd**

#found genes	0	0	0	1	2	10	18	38	99	78
FDR	0.015	0.119	0.049	0.210	0.104	0.292	0.063	0.170	0.062	0.091
APR	0.000	0.000	0.000	0.001	0.003	0.019	0.033	0.070	0.185	0.146
	0.036	0.038	0.032	0.067	0.004	0.022	0.030	0.063	0.168	0.142
(S)	0.038	0.047	0.034	0.090	0.005	0.030	0.032	0.082	0.177	0.182
(L)	0.292	0.403	0.441	0.221	0.409	0.115	0.511	0.125	0.623	0.123

	**6**		**7**		**8**		**9**		**10**	
					
	**mean**	**sd**	**mean**	**sd**	**mean**	**sd**	**mean**	**sd**	**mean**	**sd**

#found genes	215	70	284	49	318	38	333	31	332	27
FDR	0.056	0.030	0.056	0.017	0.055	0.015	0.055	0.014	0.055	0.014
APR	0.406	0.132	0.537	0.092	0.601	0.070	0.629	0.058	0.627	0.049
	0.362	0.152	0.471	0.139	0.526	0.142	0.549	0.147	0.551	0.153
(S)	0.373	0.220	0.486	0.224	0.522	0.215	0.539	0.217	0.543	0.221
(L)	0.702	0.119	0.746	0.114	0.765	0.109	0.774	0.113	0.772	0.113

The descriptive values for each interim analysis including the number detected genes, the simulated *FDR*, the real *APR*, and the *APR *estimates, all in the setting of *τ *= 5% altered genes and ten interim analyses.

#### Average Power Rate and Early Stopping

In Figure 5, the estimated and true *APR *at each interim analysis is shown. The corresponding descriptive values can be found in Tables 1 and 2. Again, the cases of *M *= 4 and *M *= 10 analyses are displayed, where half of the analyses took place during the recruitment part of the study and the other half during the follow-up part. In both cases, the estimated and the true *APR *increases with each interim analysis. In addition, true and estimated power do not diverge dramatically, however our estimation () appears to be slightly liberal. Comparable performs the estimator  (*S*), where we plug in the *π*_0 _estimation procedure of Storey and Tibshirani [8]. The power estimation by Marot and Mayer [4] ( (*L*)) overestimates the real power.

The pre-specified stopping criterion, an estimated *APR *of 80%, is represented by the dashed line. At average, this criterion is achieved at the 3rd analysis in the case of *M *= 4 planned interim analyses, and at the 7th analysis in the case of *M *= 10 planned interim analyses. In addition, true and estimated *APR *become not much higher than the desired level of 80%. In particular, the power increases when new samples are included during the recruitment part, but nearly stagnates in the follow-up part, where survival data is up-dated only.

**Figure 7:**
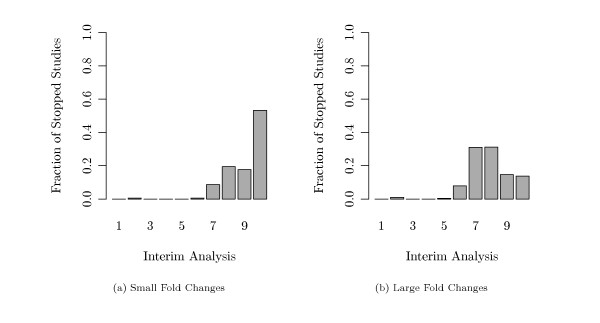
**Simulated Stopped Studies (*τ *= 50%)**. Fractions of studies that could be stopped at each interim or at the final analysis in the setting where 50% of all genes were altered between both groups. (a) Case of small fold changes, (b) case of large fold changes between the genes of short and long time survivors.

One main interest of our simulation was to find out whether interim analyses can provide an early stopping in such survival studies. Figure 7 shows for each interim analysis the fraction of simulation runs which could be stopped at this point. Both figures show the simulations with *M *= 10 analyses. The fold changes in these two settings were generated either with small effects (fold changes) or with large effects (compare Figure 3). In the case of small effects (Figure 7(a)), only 60% of all simulated studies reached the last planned final analysis while 40% were stopped at an interim analysis. In the case of large effects (Figure 7(b)), even more than 80% of the simulated studies were stopped before the final analysis.

The average power rate and its estimations in the harder setting with only *τ *= 5% survival related genes is shown in Figure 6(b). In this setting neither the true *APR *nor its estimates reach the 80% level, such that no study was stopped at earlier analyses. While the *APR *(*L*) again overestimates the true *APR*, the other estimators become conservative.

### Application to Breast Cancer Data

In order to evaluate our method on real data, we analyzed gene expression levels from 295 patients suffering from breast cancer [9]. The data contain expression levels of 24496 genes. In this study, patients were recruited between 1984 and 1995. Thus, the recruitment part of the study was *l*_1 _= 11 years. Exact arrival times were not given in the public available data set, thus, we drew these times randomly from a uniform distribution *U*(0, *l*_1_). To account for random effects, which might have been introduced by drawing the arrival times, we repeated the simulated study based on this data 1000 times with newly drawn arrival times and took the average of the resulting error and power estimations.

In the data, minimum and maximum survival was 0.5 and 18 years, respectively. Median survival was 7 years. We analyzed the data set with *M*_1 _= 5 interim analyses during the recruitment part of the study and *M*_2 _= 5 analyses within the follow-up part. We intended to control the *FDR *at a level of 5% and to stop the study when the estimated *APR *exceeds 50%.

The raw *p*-values from the final analysis are displayed in Figure 2. From this figure it can be seen, that the suggested [8] choice of *ϑ *= 0.5 is indeed a good choice for this data, as the histogram resembles a uniform distribution very well in the range [0.5, 1].

**Figure 8:**
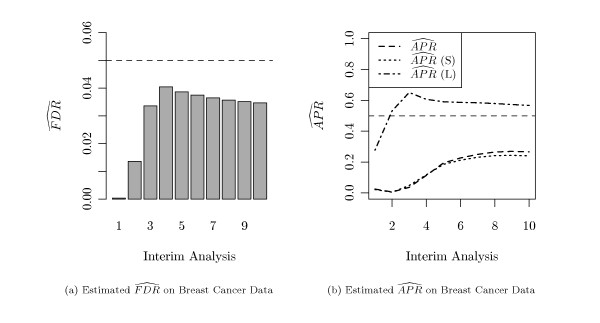
**Estimated Error and Power Rates on Real Data**. Estimated *FDR *(a) and *APR *(b) at each interim analysis of the breast cancer data.

Figure 8 shows the estimated *FDR *and estimated *APR *at each interim analysis. The pre-specified *FDR*-level of 5% is not exceeded, and the estimated *APR *increases with each interim analysis. As in the simulation study, the estimator  (*L*) seems to overestimate the real *APR*. The estimators  and  (*S*) again perform comparably. In the first interim analysis, no significant genes are detected. Thus,  and  are equal zero. Interestingly, the stopping of an estimated 50%-*APR *is not achieved in any interim analysis when the more reliable *APR *estimators are used. In detail, the maximum achieved  was only 27% (with 1900 detected genes). Therefore, the study could not be stopped early with this criterion.

Figure 8(b) illustrates the different character of recruitment and follow-up part of the study. The increase of power is much stronger in the recruitment part than during the follow-up part, meaning that in this study not the available survival data but the sample size was the more restraining factor.

### Simulation with Parameters from Breast Cancer Data

In order to perform the simulation also with different distributional assumptions, we performed an additional simulation, where parameters were taken from the breast cancer data described in the previous section. To this end we simulated the patient data and the gene expression data for 295 patients. Because the recruitment time of the real study was 11 years, the arrival times were again drawn randomly from a uniform distribution *U*(0, 11). A weibull, a gamma, and a log-normal distribution were fitted to the survival times of the real data using the fitdistcens function from the fitdistrplus R package [21]. We employed the Akaike Information Criterion (AIC) to select the fitted log-normal distribution, from which, thus, the survival times were drawn.

We wanted to set the proportion of survival related genes according to the real data set. We, therefore, used the results from the previous section where in the last analysis 1900 genes were found and the *APR *was estimated to be 27%. Thus, the total number of survival related genes in this data set was estimated to be 7037.

To generate the gene expression data, we divided the patients - as before - into two groups along their median survival time. The gene expression data was again multivariate normally distributed in both groups. The mean vector was set to 0 in one group. For the other group we used a discretization of the difference between the empirical mean vectors in both groups of the real data set. We chose the discretization grid to consist of steps with a width of 0.04, which resulted in 8542 survival related genes.

The distribution of the resulting mean vector of the second group is given in Table 4.

**Table 4:** The Mean Gene Expression Vector in the Group of Long Time Survivors

**value**											
	**-0.24**	**-0.2**	**-0.16**	**-0.12**	**-0.08**	**-0.04**	**0.00**	**0.04**	**0.08**	**0.12**	**0.16**
	
# genes	2	5	6	44	249	3336	15954	4507	335	50	8

In the simulation based on the parameters from the breast cancer data the mean vector of the gene expressions of the long time survivors is set to this discretized version of the difference of the empirical mean of the two groups in the real data.

We estimated the covariance matrix as proposed by Schäfer and Strimmer [22] using the implementation in the R package corpcor, but that resulted in a maximum power of 2.6% in the final analysis.

Therefore, we employed again a covariance matrix based on equation (12) as it was used in the other simulations. Because the effects in this simulation (see Table 4) were rather small, we reduced the simulated variance by a factor of 10 compared to the previous simulations.

**Figure 9:**
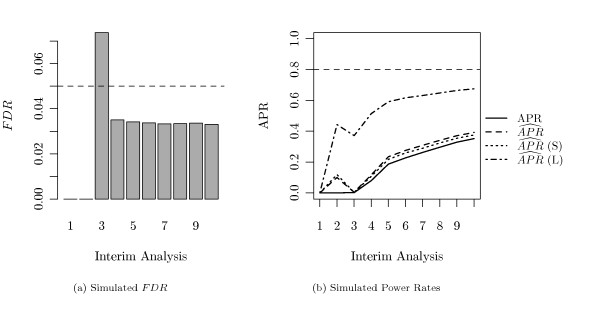
**Simulated *FDR *and Power Rate (Parameters from the Breast Cancer Data)**. The simulated *FDR *(a) and the real *APR *(solid line) with the *APR *estimates (broken lines) (b) at each interim analysis in the simulation setting where parameters were taken from the breast cancer data.

The results are shown in Figure 9 and Table 5. As in the simulation setting with *τ *= 5% survival related genes, the *FDR *peaks at the 3rd interim analysis, where only 14 genes were found on average. The *APR *estimators are comparable to the *APR *estimators in the real data, but show an erroneous aberration in the second analysis, even though there are no genes detected in that analysis. This aberration is corrected, though, in the third analysis as soon as there are some genes found.

**Table 5:** Simulated *FDR *and Power Rates (Parameters from the Breast Cancer Data)

	Analysis									
	
	1		2		3		4		5	
					
	mean	sd	mean	sd	mean	sd	mean	sd	mean	sd
#found genes	0	0	0	0	14	34	701	227	1650	396
FDR	0.000	0.000	0.000	0.000	0.074	0.179	0.035	0.008	0.034	0.005
APR	0.000	0.000	0.000	0.000	0.002	0.004	0.079	0.026	0.186	0.045
	0.000	0.000	0.100	0.160	0.004	0.008	0.117	0.031	0.234	0.044
(S)	0.000	0.000	0.118	0.238	0.003	0.007	0.107	0.029	0.217	0.043
(L)	0.000	0.000	0.443	0.374	0.371	0.032	0.514	0.030	0.591	0.029

	**6**		**7**		**8**		**9**		**10**	
					
	**mean**	**sd**	**mean**	**sd**	**mean**	**sd**	**mean**	**sd**	**mean**	**sd**

#found genes	2005	413	2318	457	2614	442	2906	447	3107	439
FDR	0.034	0.005	0.033	0.005	0.033	0.005	0.034	0.004	0.033	0.004
APR	0.227	0.046	0.262	0.051	0.296	0.050	0.329	0.050	0.352	0.050
	0.275	0.046	0.308	0.050	0.341	0.048	0.371	0.048	0.392	0.046
(S)	0.259	0.046	0.289	0.051	0.324	0.049	0.354	0.050	0.375	0.051
(L)	0.617	0.029	0.631	0.029	0.648	0.029	0.664	0.030	0.675	0.032

The descriptive values for each interim analysis including the number detected genes, the simulated *FDR*, the real *APR*, and the *APR *estimates, all in the setting where parameters were taken from the breast cancer data.

**Table 6:** Mathematical Notation

1. Patient Data	
*N*	samples
	anticipated length of the recruitment part of the study
**a **= (*a*_1_, ..., *a_N_*)	arrival times
	where *a_i _*~ *U*(0, *l*_1_), *i *= 1, ..., *N*
*l*_1 _= *max*(**a**)	length of the recruitment part
*l*_2_	length of the follow-up part
*L *= *l*_1 _+ *l*_2_	study length
*M*_1_	number of analyses within *l*_1_
*M*_2_	number of analyses within *l*_2_
*M *= *M*_1 _+ *M*_2_	number of analyses
**t **= (*t*_1_, ..., *t_N_*)	analysis times
λ	mean survival time
**s **= (*s*_1_, ..., *s_N_*)	survival times
	where *s_i _*~ *Exp*(*λ*), *i *= 1, ..., *N*
	censor variable
	where
	
2. Gene Expression Data	
*d*	genes
*d*_0_	genes not associated with survival
*d*_1_	genes associated with survival
*R*	number of positive test decisions
*FN*	false negative test decisions
*TN*	true negative test decisions
*FP*	false positive test decisions
*TP*	true positive test decisions
*τ*	fraction of differentially expressed genes
	gene expression data
*ϑ*	tuning parameter within the estimation of the proportion of true null hypotheses
*α^BH^*	the level for the type I error that is adjusted according to the BH- procedure

## Discussion

Typically, survival studies require long time spans from recruitment of the first patients until the availability of first results. Therefore, there is a strong desire to obtain results prior to the planned end of the study, not only for financial aspects but also for ethical ones. Classical group sequential designs exhibit a methodology for interim analyses including the potential for an early stopping of a trial. Whereas these classical methods concentrate on studies with one single feature, there has little been done for the case of multiple features, particularly the high-dimensional case. However, many survival studies now concentrate on correlating observed survival times with high-throughput data from genomics or proteomics experiments which yield expression levels for thousands of features measured in a small number of samples.

Based on the findings of Marot and Mayer [4] and Posch et al. [5] we simulated the possibility of early stopping in interim analyses of survival data in microarray experiments. Likewise to these prior findings we observed that a pre-specified false discovery rate is maintained during interim analyses without particular adjustments. I.e., adjustment appears only to be necessary for multiple testing but not additionally for interim analysis. While it was shown in the two mentioned articles that this principle holds asymptotically when the number of tested hypothesis is large, we have seen in further simulations beyond those presented in the section on the simulation that it also works for rather small numbers of hypotheses (e.g. testing 500 genes).

We used the Benjamini-Hochberg procedure to do the multiple testing adjustment, even though the Benjamini-Hochberg procedure does not control the *FDR *under arbitrary dependency structures. However, in our simulations and in the real data example it could be seen that this procedure mostly controlled the *FDR*. We believe, that in microarray studies a strict control of the *FDR *is of minor importance, as microarray studies are mainly used for hypothesis generation and, thus, need further validation anyway. In cases where a stricter control of the *FDR *is required, the more conservative procedure of Benjamini and Yekutieli [23] might be more appropriate.

An important issue in interim analyses of high-dimensional data is the choice of an adequate stopping criterion. Here, we chose the achieved average power rate as stopping criterion which is defined as the proportion of detected false null hypothesis. We derived a new estimator for the average power rate that comes close to the true proportion of true positive findings. However, this estimator behaved slightly liberal when the data contained many survival related genes and conservative when the data contained few survival related genes. We also tried other methods like the more sophisticated *ϑ *estimator given in [8] and the *APR *estimation method proposed in [4] which resulted in comparable and worse approximations, respectively. Improvements remain therefore necessary. With this criterion we observed that early stopping can be achieved in certain studies, based on the actual proportion of false null hypothesis and the effect sizes (size of fold changes).

**Figure 10:**
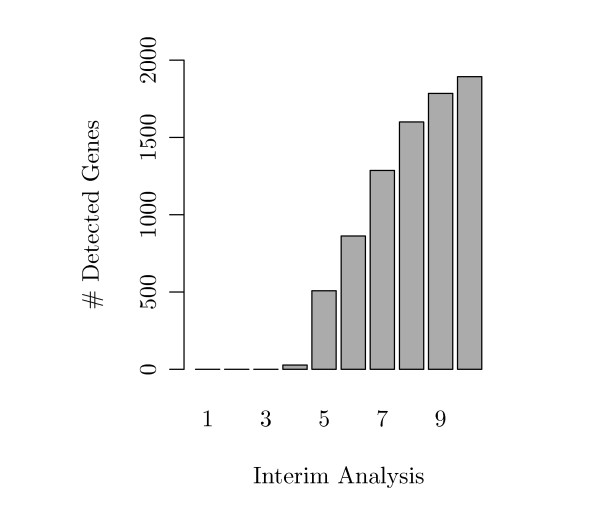
**Number of Significant Genes in Real Data**. Number of detected survival related genes at each interim analysis of the breast cancer data.

We applied the methods onto gene expression data from a microarray study on breast cancer. In this analysis we obtained an estimated average power of 20% at the fifth interim analysis (i.e. roughly five years after begin of the study) and of 27% at the eighth interim analysis (i.e., roughly after eight years). These estimated proportions seem to be rather small. However, the estimated *APR *of 27% in the final analysis corresponds to about 1900 genes detected by Cox regression (see Figure 10). This set might provide a signature which enables to build a survival predictor of sufficient quality. Predictor quality and classification accuracy are other interesting stopping criteria for interim analyses of high-dimensional data. The prognostic value of survival models based on gene expression signatures was for example studied by Hielscher et al. [24]. Evaluation of such alternative stopping criteria remains an open point which we are going to study in our further research.

The necessary sample size another important point in planning microarray studies in combination with survival data. Our simulation study provides the basis for such sample size considerations. With certain information from pilot studies, including expected distributions of fold changes and expected survival times, our simulation approach can be used to study the development of the *APR *in interim analyses. Therefore, we made our R-code available as package on the R-CRAN repository http://cran.r-project.org within the package survGenesInterim.

Several extensions of our simulation framework can be considered. In the analysis of microarray experiments normalization is an important pre-processing step to make the single arrays comparable. We therefore intent as methodological improvement to add different normalization approaches to our simulation framework. When making interim analyses, one can for example consider a re-normalization with each set of new array data or use the normalization parameters obtained in a previous interim analysis. Another improvement can be considered with regard to the survival analysis. While we have used the proportional hazard model, here, this assumptions may not always be true and other models with time variant effects seem to be more reliable.

## Conclusions

Group sequential interim analyses of microarray experiments in survival studies are frequently performed without considering the adherence of the overall error rate. Our simulation framework helps to evaluate the behaviour of error rates and power rates in such experiments. The framework also enables to study the developing of results when survival data is up-dated at subsequent times during studies that take several years.

## Authors' contributions

KJ formulated the problem and the study design. AL wrote the R-code and performed the simulations and the data evaluation. TB provided important contributions concerning the practical aspects of the study design. All authors were involved in writing the manuscript and have read and approved the final manuscript.
